# Therapeutic approaches for improving cognitive function in the aging brain

**DOI:** 10.3389/fnins.2022.1060556

**Published:** 2022-12-08

**Authors:** Lingmin Chen, Jiao Jiao, Yonggang Zhang

**Affiliations:** ^1^Department of Anesthesiology and National Clinical Research Center for Geriatrics, West China Hospital, Sichuan University and The Research Units of West China (2018RU012), Chinese Academy of Medical Sciences, Chengdu, China; ^2^Department of Periodical Press and National Clinical Research Center for Geriatrics, West China Hospital, Sichuan University, Chengdu, China

**Keywords:** aging, Alzheimer’s disease, dementia, mild cognitive impairment, pharmacological, non-pharmacological, intervention

## Abstract

The rapid aging of populations around the world has become an unprecedented challenge. Aging is associated with cognitive impairment, including dementia and mild cognitive impairment. Successful drug development for improving or maintaining cognition in the elderly is critically important. Although 4 drugs for improving cognition in Alzheimer’s disease have been approved, a variety of potential drugs targeting age-related cognitive impairment are still in development. In addition, non-pharmacological interventions, including cognition-oriented treatments, non-invasive brain stimulation physical exercise, and lifestyle-related interventions, have also been suggested as cognitive enhancers in the last decade. In this paper, we reviewed the recent evidence of pharmacological and non-pharmacological interventions aimed at improving or maintaining cognition in the elderly.

## Introduction

The world’s population is aging. The population aged 65 and above reached 398 million in 2020, about 9.3% in the total population (United Nations, 2019), and will reach 2 billion by 2050 (Clegg et al., 2013). Increased age is associated with cognitive impairment, including dementia and mild cognitive impairment (MCI). Dementia is characterized by the decline of memory, thinking, perception, language, decision-making, planning, and reasoning (Power et al., 2019). Globally, almost 50 million population are dementia, and the number will increase to 152 million by 2050 (World Health Organization, 2019). Dementia increases with age and is about 5.0% for people between 71 and 79 years old, 24.2% for people between 80 and 89 years old, and 34.7% for people aged 90 years or older (Plassman et al., 2007). Among dementia, Alzheimer’s disease (AD) is the most common type (World Health Organization, 2019). MCI is a stage between normal cognitive aging and dementia (Power et al., 2019), and it is about 10–20% in the population aged 65 years and above according to different diagnostic criteria, and it increases with age (Langa and Levine, 2014). MCI is assumed to be a precursor of dementia; MCI individuals have higher risk of dementia, and about 14.9% MCI elderly will be dementia in 2 years (Morley, 2018; Petersen et al., 2018).

Dementia could cause disability and dependency and results in a huge economic and social burden (Wimo et al., 2017). Therefore, there is a growing need to identify the mechanisms of aging-related cognitive impairment with a perspective on potential targets and explore interventions that can improve or maintain cognition and delay the progression of dementia. In the current study, we reviewed mechanisms of aging and cognitive impairment and summarized evidence of both pharmacological and non-pharmacological interventions and hoped to provide evidence for future studies.

## Aging and cognitive impairment

As reported previously (Satoh et al., 2017), aging will result in the decline of cognitive function and alterations in brain structure. It leads to brain atrophy, especially in the hippocampus and prefrontal cortex (Satoh et al., 2017). Brain atrophy is related to age-related neuronal loss, reduced neurogenesis, and reduction in dendritic branching and dendritic spines. Aging also affects synapse density and synaptic function, leading a reduced synaptic transmission and plasticity (Bettio et al., 2017). Numerous factors contribute to structural and functional changes. Aging leads to intracellular accumulation of ROS, causing damage to cellular macromolecules and mitochondria (Castelli et al., 2019). Aging also induces microglia and astrocyte activation, leading to excessive neuroinflammation and consequent neuronal damage (Clarke et al., 2018; Edler et al., 2021). Amyloid-β deposition and tau aggregation have also been found in the aging brain (Zhang et al., 2021). Neurotrophins are associated with neurogenesis, neuronal survival, and synaptic plasticity (Sardar et al., 2021). Disruption of brain-derived neurotrophic factor (BDNF) and vascular endothelial-derived growth factor (VEGF) signaling in the aging brain was also associated with cognitive deficits (Bettio et al., 2017). Another factor to consider about the aging brain and impaired cognition is the decreased level of neurotransmitters, such as dopamine and serotonin (Peters, 2006). Aging contributes to cognitive decline, resulting in healthy individuals to neurodegenerative diseases, particularly AD. An increasing number of studies suggest normal aging and neurodegenerative disorders share similar functional, structural, and cellular changes. However, the structural brain changes are much more significant in neurodegenerative disorders (Yankner et al., 2008; Bettio et al., 2017).

## Pharmacological interventions for cognition improvement in the aging brain

Successful drug development for improving or maintaining cognition in seniors is critically important. Although many novel targets are being explored for improving cognition in the past two decades, there are only several drugs approved to improve cognition in AD and, no drug has been approved for cognitive protection in MCI patients (Fink et al., 2018; Petersen et al., 2018). Here, we introduced the clinical evidence on the approved drugs and promising drug targets (Table 1).

**TABLE 1:** Evidence of pharmacological interventions for cognitive impairment in older adults.

Study	Condition	Study design	Intervention	Main results
Birks and Harvey, 2018	AD	Meta-analysis, including 30 studies (8,257 participants)	Donepezil	Small benefits in cognitive function; Higher dose involved with higher withdrawal and of adverse events
Birks and Grimley Evans, 2015	AD	Meta-analysis, including 30 studies (3,450 participants)	Rivastigmine	Benefits for cognitive function.
Jiang et al., 2015	AD	Meta-analysis, including 11 studies (4,074 participants)	Galantamine	Improved cognitive function.
Birks, 2006	Dementia due to AD	Meta-analysis, including 13 studies (7,298 participants)	Donepezil, galantamine and rivastigmine	All efficacies and no difference between drugs. Fewer adverse events in donepezil compared with rivastigmine.
Battle et al., 2021	Vascular dementia, vascular cognitive impairment	Meta-analysis, including 8 studies (4,373 participants)	Donepezil, galantamine, and rivastigmine	Donepezil, and galantamine had beneficial effect with not clinically important, while rivastigmine was less certain.
Matsunaga et al., 2019	MCI	Meta-analysis, including 14 studies (5,278 participants)	Donepezil, galantamine, and rivastigmine	No effect on cognitive function, might decrease progression to dementia, fall, and increase discontinuation.
McShane et al., 2019	Dementia (AD, vascular dementia)	Meta-analysis, including 44 studies (almost 10,000 participants)	Memantine	Benefit in moderate-to-severe AD, but not in mild AD.
Reger et al., 2006	Early AD and MCI	Trial (61 participants)	Intranasal insulin	Improved verbal memory.
Reger et al., 2008a	Early AD and MCI	RCT (24 participants)	Intranasal insulin	Retained more verbal information, improved attention, and functional status.
Reger et al., 2008b	AD and MCI	Trial (92 participants)	Intranasal insulin	Facilitated recall on verbal memory in different genotype of APOE.
Craft et al., 2012	Mild to moderate AD and MCI	RCT (104 participants)	Intranasal insulin	Improved delayed memory.
Campbell et al., 2018	Diabetes	Meta-analysis, including 14 studies	Metformin	Reduced cognitive impairment and dementia incidence
Luchsinger et al., 2016	MCI	RCT (80 participants)	Metformin	Reduced the change in total recall; Adverse reaction of gastrointestinal symptoms.
Koenig et al., 2017	AD	RCT (20 participants)	Metformin	Improved executive function, with learning/memory and attention.
Gauthier et al., 2015	Mild-to-moderate AD	Meta-analysis, including 6 studies (810 participants)	Cerebrolysin	Beneficial and safety
Guekht et al., 2011	Vascular dementia	RCT (242 participants)	Combination of cerebrolysin and acetylsalicylic acid	Improved cognitive outcome with safe and well tolerated.
Alvarez et al., 2011	Mild-to-moderate AD	RCT (197 participants)	Cerebrolysin, donepezil and a combination of both treatments	Cerebrolysin is equally to donepezil. Combined therapy showed better cognitive improvement.

### Cholinesterase inhibitors

Acetylcholine is associated with memory, learning, attention, and other essential aspects of cognition (Hampel et al., 2018). Aging and AD are associated with cholinergic neuron loss (Terry and Buccafusco, 2003; Haam and Yakel, 2017). Cholinesterase inhibitors (ChEIs) block acetylcholinesterase, thereby delaying the breakdown of acetylcholine and enhancing cholinergic neurotransmission. ChEIs, including donepezil, galantamine, and rivastigmine, have been approved for treating AD (Oh and Rabins, 2019). Donepezil is a non-competitive acetylcholinesterase inhibitor that was approved in 1996. Donepezil administration for 12 or 24 weeks has a small beneficial improvement on cognition in AD. The beneficial effects of 10 or 23 mg daily are slightly larger than 5 mg daily. However, the rates of withdrawal and adverse events are higher with higher doses (Birks and Harvey, 2018). Rivastigmine can inhibit both acetylcholinesterase and butyrylcholinesterase and was approved in 2000. Rivastigmine could also improve cognitive function in AD (Birks and Grimley Evans, 2015). Galantamine is a competitive inhibitor of acetylcholinesterase. Administration with 16–40 mg galantamine daily for 8–28 weeks can significantly enhance cognitive function (Jiang et al., 2015). Although there are slight variations in the mode of actions, there are no differences in their efficacy for cognitive improvement in AD (Birks, 2006). A network meta-analysis assessed the efficacy of cholinesterase inhibitor for vascular dementia and other vascular cognitive impairments, donepezil, and galantamine were found to slightly improve cognition, although the effects were not clinically significant. The evidence for rivastigmine is less certain (Battle et al., 2021). A meta-analysis evaluating the clinical benefit of ChEIs for MCI has been published in 2019 by Matsunaga et al. (2019). That study included 14 randomized controlled trials (RCTs) with 5,278 subjects, where 6 studies involved donepezil, 4 involved galantamine, and 4 involved rivastigmine. The results found that ChEIs did not improve cognitive function in MCI adults, however, ChEIs significantly slowed down the progression of dementia. As there are higher incidences of discontinuation due to adverse events, including nausea, vomiting, abnormal dreams, diarrhea, dizziness, bradyarrhythmia, syncope, and weight loss, ChEIs are not recommended for improving MCI (Matsunaga et al., 2019; Oh and Rabins, 2019). New versions of ChEIs such as octohydroaminoacridine and AD-35 are in development (Cummings et al., 2021).

### Memantine

Besides cholinesterase inhibitors, memantine, a low-affinity NMDA receptor antagonist, has also been approved for treating AD (Huang and Mucke, 2012). In a Cochrane review, the clinical benefit of memantine was found in moderate-to-severe AD. However, there was no clinical benefit of memantine for mild AD. Whether a long duration of memantine administration is beneficial for mild AD needs to be assessed. There is limited evidence of memantine for MCI and other causes of dementia (Ferris et al., 2007; McShane et al., 2019). Currently, SAGE-718, another NMDA receptor positive allosteric modulator, is in Phase II clinical trial (Cummings et al., 2021).

### Antidiabetic agents

Diabetes is another risk factor for dementia, and diabetes increase a 1.5–2 times higher risk of dementia (Areosa Sastre et al., 2017). Association between aging, dementia with disruption of insulin receptor signaling has been reported, insulin resistance has also been proposed as a mechanism for cognitive impairment. Restoration of insulin signaling in the brain can be a potential way to improve cognition (Boccardi et al., 2019; Erichsen et al., 2021).

#### Intranasal insulin

Intranasal administration of insulin can increase insulin levels in the central nervous system (de la Monte, 2013; Erichsen et al., 2021). Previous studies demonstrated that intranasal insulin had potentially beneficial effects on cognitive functions (Reger et al., 2006, 2008a,b). Acute intranasal insulin (20 IU) treatment facilitates verbal memory in the elderly with MCI or AD, and these effects were stronger for patients without the APOE-epsilon4 allele (Reger et al., 2006, 2008a,b). Craft et al. (2012) reported chronic intranasal insulin therapy could preserve general cognition and improve delayed memory in MCI and AD patients. These findings indicate the potential beneficial effect of insulin on cognitive decline.

#### Metformin

Metformin is a first-line antihyperglycemic drug and works by increasing insulin sensitivity in peripheral tissues and suppressing hepatic gluconeogenesis (Boccardi et al., 2019). Although a meta-analysis of 6 cohort studies has shown metformin might reduce the incidence of dementia in diabetic patients (Campbell et al., 2018), the evidence of metformin usage in non-diabetic adults is still limited. In a pilot randomized controlled trial with 80 MCI adults, metformin was found to increase verbal memory. Metformin was tolerated by 92.5% of participants, and no serious adverse events occurred (Luchsinger et al., 2016). Another study found that metformin administration for 8 weeks was associated with improved executive functioning in patients with MCI or mild dementia (Koenig et al., 2017), which revealed the potential beneficial effect of metformin in the elderly.

### Cerebrolysin

Cerebrolysin is a mixture of neuropeptide and amino acids produced by the enzymatic breakdown of pig’s brain tissue, which acts as endogenous neurotrophic factors (Cui et al., 2019). Currently, cerebrolysin is used as the treatment for dementia in Europe and Asia (Cui et al., 2019; Gavrilova and Alvarez, 2021). In a meta-analysis, cerebrolysin was suggested to have beneficial effects on cognitive function in mild-to-moderate AD (Gauthier et al., 2015). The beneficial effect of cerebrolysin on cognitive function was also found in vascular dementia (Guekht et al., 2011). Alvarez et al. (2011) compared cerebrolysin and donepezil, and found that cerebrolysin was as effective as donepezil in cognitive improvement, and the combinational therapy had better cognitive performance than single-drug treatment. In addition, whether cerebrolysin administration has a beneficial effect on cognitive function in cognitive healthy aging adults and in patients with MCI is still inconclusive, and whether cerebrolysin administration can delay the progression of dementia also needs to be further analyzed.

### Potential drugs with novel mechanisms

Drug development for AD has been challenging for the last two decades, although a variety of potential drug targets have been identified (Cummings, 2021; van Bokhoven et al., 2021). No cognitive enhancing agent for AD has been recently approved for cognitive improvement in AD (Cummings, 2021; van Bokhoven et al., 2021). Table 2 shows the 13 drugs in clinical trials for the treatment of cognitive. Among them, one drug is in phase I trial, 6 drugs are in phase II trial, and 6 drugs are in phase III trial (Cummings et al., 2021).

**TABLE 2:** Clinical trials target cognitive enhancement for Alzheimer’s disease (AD) patients in 2021 in Clinicaltrials.gov.

NCT number	Phases	Interventions	Status	Conditions	Locations	Gender	Age (years)	Enrollment	Funder
NCT04308304	Phase 1	Drug: MK-1942; Drug: Donepezil; Drug: Placebo	Completed	AD	USA	All	50–85	27	Industry
NCT04249869	Phase 1, Phase 2	Drug: VGH-AD1	Unknown status	AD	China	All	≥65	28	Other
NCT04602624	Phase 2	Drug: SAGE-718	Completed	AD,CD,MD	USA	All	50–80	26	Industry
NCT02720445	Phase 2	Drug: Nicotine transdermal patch; Drug: Placebo patch	Recruiting	MCI	USA	All	55–90	380	Other| NIH
NCT04044131	Phase 2	Drug: Metabolic cofactor supplementation; Drug: Sorbitol	Unknown status	AD	Turkey	All	≥18	120	Other| Industry
NCT04601038	Phase 2	Drug: CORT108297; Drug: Placebo	Recruiting	AD,MCI	USA	All	≥55	52	Other
NCT03625401	Phase 2	Drug: AD-35 60 mg group; Drug: Placebo group	Unknown status	AD	China	All	50–85	55	Industry
NCT03090516	Phase 2, Phase 3	Drug: Ginkgo biloba dispersible tablets; Drug: Donepezil; Drug: Ginkgo biloba dispersible tablets and donepezil	Unknown status	AD	China	All	50–85	240	Other
NCT04229927	Phase 3	Drug: BPDO-1603	Recruiting	AD	China	All	≥45	712	Industry
NCT04661280	Phase 3	Drug: Donepezil	Recruiting	AD	France	All	≥50	240	Other
NCT04570085	Phase 3	Drug: Caffeine| Drug: Placebo	Recruiting	AD	France	All	≥50	248	Other
NCT03283059	Phase 3	Drug: Octohydroaminoacridine succinate; Drug: Aricept; Drug: Placebos	Unknown status	AD	China	All	50–85	600	Other, industry
NCT03116126	Phase 3	Drug: Guanfacine; Drug: Placebo	Recruiting	AD	UK	All	≥45	160	Other

#### Phosphodiesterases inhibitors

Phosphodiesterases (PDEs) are a superfamily (including 11 isoforms) that can catalyze second messengers cAMP and cGMP which have important roles in learning and memory (Wu et al., 2018). Almost all PDE isoforms are mostly expressed in the brain, especially in learning and memory regions (Wu et al., 2018). PDEs inhibitors showed remarkable cognitive enhancement in preclinical studies (Bruno et al., 2011; Peters et al., 2014; Zhang et al., 2018), and some PDE inhibitors have been tested in clinical trials. Most showed good safety and tolerability in phase I trial, and over 10 are in phase II/III/IV trials. Vinpocetine is a classical PDE1 inhibitor and was discovered about 40 years ago (Prickaerts et al., 2017). Though vinpocetine was found effect in improving learning and memory in preclinical study, vinpocetine didn’t show beneficial effect on cognition in AD patients (Szatmari and Whitehouse, 2003). Cilostazol is a selective PDE3 inhibitor. Cilostazol coadministration with donepezil or galantamine ameliorated cognitive decline efficiently in patients with moderate AD (Arai and Takahashi, 2009; Hishikawa et al., 2017). A cohort study recruiting 9148 participants found cilostazol treatment reduced the risk of developing dementia (Tai et al., 2017). Roflumilast is a selective PDE4 Inhibitor. Roflumilast has completed the phase II trial as a cognition enhancer in healthy adults (Van Duinen et al., 2018). PF-04447943 is a selective PDE9A inhibitor. Although PF-04447943 was safe and well-tolerated, PF-04447943 administration did not affect cognition when compared to the placebo (Schwam et al., 2014).

#### Muscarinic and nicotinic acetylcholine receptor agonists

Besides ChEIs, mAChRs agonists and nAChRs agonists are being tested as the other two promising drug targets for cognitive improvement in AD. Xanomeline, the first generation mAChRs agonist, has been demonstrated to improve cognition in a phase III trial. However, due to the relatively low M1 receptor selectivity, xanomeline can lead to cholinergic adverse effects such as sweating, salivation, and gastrointestinal disturbances because of activating peripheral M2 and M3 mAChRs (Scarpa et al., 2020). Unfortunately, later developed highly selective M1 mAChRs agonists, such as PF-06767832, MK-7622, and PF-06764427, lead to cholinergic toxicity and behavioral convulsions (Davoren et al., 2016; Voss et al., 2018). nAChR agonists are less developed than mAChR agonists. There are several nAChR agonists developed for the cognitive improvement of AD patients in the past two decades, such as encenicline (EVP-6124) and TC-1734 (AZD-3480). Encenicline is well tolerated at single doses in healthy volunteers. However, it was later suspended in the phase III trial due to serious gastrointestinal adverse effects in elderly patients (Hoskin et al., 2019). TC-1734 is a selective α4β2 nAChR agonist (Dunbar et al., 2011). A randomized placebo-controlled trial investigated its effects on cognition. Compared to the placebo group, patients in TC-1734 (50 mg) groups showed superior performance on attention and episodic memory (Dunbar et al., 2011). In addition, a phase II trial analyzed its effect on cognitive function in MCI adults (Cummings et al., 2021). mAChRs agonist and nAChRs agonist could be promising treatments. However, there is still a long way to go before advancing them into the market (Verma et al., 2018).

#### Dopamine agonist

Decreased release of dopamine and decreased expression of dopamine receptors are associated with age-related cognitive decline (Volkow et al., 2000). There is evidence of the involvement of dopamine in AD. Restoration of dopamine transmission can improve learning and memory of AD (Guzmán-Ramos et al., 2012; Martorana and Koch, 2014). In a trial of 60 participants, dopaminergic agonist piribedil administration improves global cognitive function in MCI patients (Nagaraja and Jayashree, 2001). Koch et al. (2020) investigated the effect of the dopaminergic agonist rotigotine on cognitive functions in mild to moderate AD, and found rotigotine did not affect global cognition; however, it could improve cognition associated with the frontal lobe. Monoamine oxidase B (MAO-B) inhibition could increase the availability of dopamine (Koch et al., 2020). In a phase II trial, Matthews et al. evaluated the potential benefit of rasagiline (a selective MAO-B inhibitor) in mild to moderate AD with 50 participants. The results showed rasagiline could improve brain metabolism as measured by fluorodeoxyglucose–positron emission tomography. However, it did not affect globe cognitive function (Matthews et al., 2021). Larger sample size trials are needed to evaluate the effect of dopaminergic stimulation on cognitive impairment.

### Cognitive enhancing drugs in healthy adults

There were also drugs enhancing cognition in health adults in elderly, potential drugs including substances acting on neurotransmitters, hormones, transduction systems, and brain perfusion and metabolism (Milić et al., 2021). Stimulants such as amphetamine and methylphenidate were reported to improve executive function and memory in healthy adult (Smith and Farah, 2011; Ilieva et al., 2015). Modafinil is an FDA-approved eugeroic that could preserve alertness under conditions of sleep deprivation, through increases cortical catecholamine levels. Most studies found modafinil could enhances executive function, attention and learning and memory (Battleday and Brem, 2015; Farah, 2015). However, their cognitive enhancing effect and safety on elder adults are still needed investigation. What’s more, these drugs are often misused and have abuse potential, which should be paid with more caution (Buccafusco, 2009; Milić et al., 2021).

## Non-pharmacological interventions for cognition improvement in the aging brain

A growing number of studies show that non-pharmacological interventions can enhance cognition in the last decade (Gavelin et al., 2020; Sikkes et al., 2021). Non-pharmacological interventions covered a diverse range of intervention categories, including cognition-oriented treatments, non-invasive brain stimulation physical exercise, and lifestyle-related interventions (Table 3). Different clinical stages of cognitive impairment, from MCI to dementia, could all benefit from non-pharmacological treatments. Most non-pharmacological treatments have few adverse effects and can be combined with pharmacological treatments (Sikkes et al., 2021).

**TABLE 3:** Evidence of non-pharmacological interventions for cognitive impairment in older adults.

Study	Interventions	Condition	Study design	Main findings
Bahar-Fuchs et al., 2019	Cognitive training	Mild to moderate dementia	Meta-analysis, including 32 studies (2,462 participants)	Positive effects on global cognition, verbal semantic fluency.
Gates et al., 2019	Computerized cognitive training	MCI	Meta-analysis, including 8 studies (660 participants)	Uncertain.
Hu et al., 2021	Computerized cognitive training	MCI and dementia	Meta-analysis, including 12 studies (participants)	Positive effect on cognitive function
Woods et al., 2012	Cognitive stimulation	Dementia	Meta-analysis, including 15 studies (718 participants)	Positive effect on cognitive function
Cai et al., 2019	Transcranial direct current stimulation	Mild to moderate AD	Meta-analysis, including 7 studies (146 participants)	Positive effect on cognitive function
Chu et al., 2021	Transcranial direct current stimulation	AD,MCI	Meta-analysis, including 27 studies (1,070 participants)	Positive effect on improving global cognition
Cheng et al., 2018	Repetitive transcranial magnetic stimulation	Mild to moderate AD	Meta-analysis, including 7 studies (194 participants)	Improved cognitive function in mild to moderate AD.
Xie et al., 2021	Repetitive transcranial magnetic stimulation	MCI and early AD	Meta-analysis, including 12 studies (4,380 participants)	Improved cognitive function in MCI and early AD. The improvement could last for 1 month and MCI patients had more benefits.
Coelho-Júnior et al., 2021	Mediterranean diet	Older adults	Meta-analysis, including 53 studies	A better global cognition and memory. No association in the incidence of mobility problems, MCI, and dementia.
Lv et al., 2021	Healthy elderly; AD; Major depressive disorder; Minimal hepatic encephalopathy; Fibromyalgia	Meta-analysis, including 7 studies (320 participants)	Probiotics	Enhanced cognitive function; Single strain is better than multiple strains.
López-Ortiz et al., 2021	Exercise	AD	Meta-analysis, including 28 studies (1,337 participants)	Benefit effect.
Jia et al., 2019	Physical activity and exercise	AD	Meta-analysis, including 13 studies (673 participants)	Improvement in cognition.
Angevaren et al., 2008	Physical activity	Older people without known cognitive impairment	Meta-analysis, including 11 studies (620 participants)	Beneficial for cognitive function.
Law et al., 2020	Physical exercise	MCI, dementia	Meta-analysis, including 46 studies (5,099 participants)	Reduced the decline in global cognition in MCI or dementia.
Sanders et al., 2019	Aerobic, anaerobic, multicomponent, or psychomotor exercise	Older adults	Meta-analysis, including 36 studies (2,007 participants)	Improved executive function and memory. Short session duration and high frequency predict a higher effect on cognitive impairments.
Chan et al., 2020	Latin, ballroom, and aerobic dances	MCI	Meta-analysis, including 5 studies (358 participants)	Improved global cognition, attention, immediate and delayed recall, and visuospatial ability.
Hewston et al., 2021	Dance	Older adults	Meta-analysis, including 11 studies (1,412 participants)	Improved global cognitive function and executive function.
Ruiz-Muelle and López-Rodríguez, 2019	Dance	AD	Meta-analysis, including 12 studies (349 participants)	Positive effect on physical and cognitive function, and quality of life.
Ngandu et al., 2015	Multi-domain intervention of diet, exercise, cognitive training, and vascular risk monitoring	Older adults	RCT (1,190 participants)	Improve or maintain cognitive functioning in at-risk elderly people from the general population.
Hoevenaar-Blom et al., 2021	A multi-domain intervention that targeted vascular risk factors (smoking, unhealthy diet, physical inactivity, overweight, hypertension, dyslipidemia, and diabetes)	Older adults	RCT (3,526 participants)	Not reduce dementia incidence in old age.
Sanders et al., 2019	Isolated supplementation with omega-3 fatty acid, an isolated multi-domain intervention (consisting of nutritional counseling, physical exercise, cognitive stimulation) or a combination of the two interventions	Older adults	RCT (1,680 participants)	No effect on the cognitive decline over 3 years was found.
Hafdi et al., 2021	Multi-domain interventions	Older adults	Meta-analysis, including 9 studies (18,452 participants)	Reducing dementia incidents, a small improvement in cognitive function.
Gavelin et al., 2021	Combined physical and cognitive training	Older adults	Meta-analysis, including 41 studies (4,052 participants)	Small effect and statistically significant for overall cognitive and physical function. Simultaneous training was best, followed by sequential combinations and cognitive training alone.
Liu et al., 2021	Nutrition and exercise interventions	MCI	Meta-analysis, including 6 studies (1,039 participants)	Improve global cognitive function. No difference in MMSE scores, memory, executive function, attention, and information processing speed across groups.

### Cognition-oriented interventions

Cognition-oriented interventions, such as cognitive training, cognitive stimulation, and cognitive rehabilitation, are approaches for the prevention and treatment of cognitive decline in the elderly (Gavelin et al., 2020). They are in high availability, high accessibility, and low implementation costs. Cognitive training consists of repeated practices on standardized tasks aimed at improving or maintaining certain aspects of cognitive functions (Bahar-Fuchs et al., 2019). The difficulty of training tasks should be adjusted according to participants’ performance, which is particularly feasible when using computerized cognitive training. Cognitive stimulation involves non-specific engagement in activities for improving cognitive status (Bahar-Fuchs et al., 2019). Cognitive rehabilitation could achieve or preserve optimal levels of functioning in daily life (Gavelin et al., 2020). Cognitive training has also been reported to have a small to moderate effect on global cognition and a moderate effect on verbal semantic fluency for mild to moderate dementia (Bahar-Fuchs et al., 2019). Gates et al. evaluated the effect of computerized cognitive training through a meta-analysis. However, no conclusion could be drawn on whether computerized cognitive training had a beneficial effect on cognitive function as the evidence was of low quality, and most of the results were imprecise (Gates et al., 2019). Hu et al. included both MCI and dementia patients with computerized cognitive training in a systematic review in 2021. They included 12 studies and found computerized cognitive training could improve general cognition, especially memory. Subgroup analysis found computerized cognitive training on cognition for dementia was almost double for MCI (Hu et al., 2021). Cognitive stimulation was also associated with improved cognitive function, self-reported quality of life, and communication and social interactions in dementia people (Woods et al., 2012). Although cognition-oriented treatments showed a positive effect on cognitive function, high-quality with larger sample size trials are needed, and further studies should be performed to address the potential benefits of longer-term interventions and their clinical significance.

### Non-invasive brain stimulation

As for non-invasive brain stimulation, transcranial electrical stimulation (TES) and transcranial magnetic stimulation (TMS) are the main techniques. Both techniques are safe and can be well tolerated without sedation or anesthesia (Brunoni et al., 2019). They both work by modulating synaptic efficacy and neural circuit and have been used in clinical practice.

#### Transcranial electrical stimulation

TES applicant a low-intensity (1–2 mA) electric current to the brain *via* two electrodes (anode and cathode), and transcranial direct current stimulation (tDCS) is the most studied. The effects of tDCS are determined by the electrical current direction. Anodal tDCS increases neuronal activities by depolarizing the resting potential, while the cathodal tDCS inhibits neuronal activities by hyperpolarizing the resting potential (Grimaldi et al., 2020). Cai et al. evaluated the effects of tDCS on cognition within mild to moderate AD patients through a meta-analysis. The results revealed that tDCS could enhance cognitive function; in addition, only a single session of tDCS was effective, repeated sessions of tDCS were not effective, and lower current density (0.06 mA/cm^2^) but not higher current density (0.08 mA/cm^2^) enhanced cognition (Cai et al., 2019). Recently, Chu et al. analyzed the cognitive effects of TES on AD and MCI. After a 1-month follow-up, cathodal tDCS revealed larger therapeutic responses than anodal tDCS on general cognitive function. Subgroup analysis only found patients with AD, but not MCI, significantly responded to cathodal tDCS (Chu et al., 2021).

#### Transcranial magnetic stimulation

Transcranial magnetic stimulation uses a magnetic field to induce action potentials. The effects of TMS are determined by stimulation frequency. When the frequency is equal to or below 1 Hz, neural excitability is decreased. When the frequency is between 5 and 20 Hz, neural excitability is increased (Cespón et al., 2018). TMS can use different stimulation patterns, including single-pulse TMS (sTMS), double (or paired) pulse TMS (dTMS), and repetitive TMS (rTMS). sTMS consists of the discharge of single pulses interleaved by at least 4 s periods off-stimulation, dTMS consists of the discharge of a test stimulus preceded by a conditioning stimulus, rTMS refers to more than two pulses delivered within a time interval of 2 s or less (Valero-Cabré et al., 2017). rTMS has been widely investigated in depression, and it has been approved by FDA for medication-resistant depression (Iriarte and George, 2018). In recent years, rTMS has been considered as a promising intervention for cognitive improvement (Iriarte and George, 2018). Two systematic reviews reported high frequency rTMS might show a moderate effect on cognition in AD and MCI patients (Cheng et al., 2018; Xie et al., 2021). However, the conclusion was limited by the small sample size of included studies. Larger RCTs and additional research are needed to identify the effect of TMS in the elderly with cognitive impairment.

### Dietary and nutrition

Nutrition is an important factor that contributes to healthy aging. Adopting a healthier diet may be beneficial to cognition (Jennings et al., 2020). World Health Organization (2019) has advocated a healthy diet to reduce the risk of cognitive decline and/or dementia. Some, but not conclusive, evidence suggests that certain nutrients are protective of brain health in the elderly, including long-chain omega-3 fatty acids, vitamin B, vitamin D, selenium and etc. (Scarmeas et al., 2018). Dietary patterns were also suggested to be protective for brain health in elderly (Scarmeas et al., 2018; Power et al., 2019; Flanagan et al., 2020). The Mediterranean diet was the most extensively studied dietary pattern (Chen et al., 2019). It involves a high intake of vegetables, fruits, legumes, olive oil, whole grains, fish, low to moderate intake of dairy products, alcohol, and restrictions on red meat (Power et al., 2019). High adherence to the Mediterranean diet is associated with better global cognition and memory has been reported by meta-analysis (Coelho-Júnior et al., 2021). However, whether it could reduce the risk of developing MCI or dementia is still conflicted (Coelho-Júnior et al., 2021; García-Casares et al., 2021). The ketogenic diet was another specific diet, which might provide treatment benefits for AD patients. However, the current studies might be limited by small sample size, short-terms effects, and future studies should be further performed (Hersant and Grossberg, 2022). The dietary intervention could be considered alongside other individualized interventions to improve cognition in elderly adults.

Interaction between gut microbes and the brain has received considerable attention in the past decade (Martin et al., 2018; Willyard, 2021). Gut microbiota is found to be associated with emotion, cognition, and social behavior (Sarkar et al., 2018). Probiotic intervention works by delivering specific strains of bacteria that increase the diversity and number of beneficial microbes, thereby altering the gut microbiota (Eastwood et al., 2021). Lv et al. evaluated the probiotics on cognition by meta-analysis, and they found that probiotic supplementations improved cognitive function. Subgroup analyses further found the enhanced effect existed only in people with impaired cognition. Furthermore, a single strain was more effective than multiple strains (Lv et al., 2021). Thus, probiotics have been suggested as an effective and accessible cognitive therapy; however, more randomized controlled clinical trials are needed for this conclusion.

### Exercise

Emerging evidence indicates exercise not only promotes physical health but also contributes to the preservation of cognition function. The mechanisms account for the neuroprotective effects of exercise on the brain include evaluated neurotrophic factor levels, increased synaptogenesis, improved vascularization, decreased systemic inflammation, and reduced abnormal protein deposition (Kirk-Sanchez and McGough, 2014). Several meta-analyses analyzed the effects of exercise on cognition, focused predominantly on aerobic exercise (Jia et al., 2019; Sanders et al., 2019; López-Ortiz et al., 2021). Angevaren et al. (2008) performed a Cochrane review in older people without known cognitive impairment. They found that aerobic exercise increased cognitive capacity, including motor function, cognitive speed, and visual attention. Another meta-analysis reported aerobic exercise attenuated the cognitive decline in MCI and dementia people, and found that working memory decline was significantly attenuated, and the effects on other domains of cognitive functions were unclear. Moderate to high-intensity aerobic exercise had a better effect on cognition (Law et al., 2020). Another meta-analysis examined the dose-response relationship and found shorter sessions and higher frequencies of exercise could generate a better cognitive effect (Sanders et al., 2019).

### Dance intervention

Dancing intervention is another strategy, because it requires physical, cognitive, and social abilities, and thus been analyzed in many studies. In a recent meta-analysis in MCI populations, the results showed that dance had a small to moderate effect on cognitive function, such as attention, immediate and delayed recall, global cognition, and visuospatial ability (Chan et al., 2020). Another meta-analysis involving both healthy and MCI old adults also found dance enhanced global cognitive function and executive function (Hewston et al., 2021). The positive effect of dance intervention on cognitive function in adults with AD was also confirmed in a systematic review (Ruiz-Muelle and López-Rodríguez, 2019). Thus, dance has been suggested as an adjunct therapy for cognitive decline in the aging population.

### Multi-domain interventions

As cognitive impairment is a complex, multifactorial disorder, multi-domain interventions have been suggested as a new strategy (Kivipelto et al., 2018). In the last decades, 3 large clinical trials with multi-domain interventions (FINGER, MAPT, and PreDIVA) have been reported. In the FINGER study, diet, exercise, cognitive training, and vascular risk monitoring were used to improve cognitive function in elderly people at risk for cognitive impairment (Ngandu et al., 2015). In the preDIVA study, a multi-domain intervention targeted vascular risk factors of smoking, unhealthy diet, physical inactivity, overweight, hypertension, dyslipidemia, and diabetes over 12 years was used. However, it did not reduce dementia risk in older people (Hoevenaar-Blom et al., 2021). In the MAPT trial, multi-domain intervention and Omega-3 PUFA supplementation were involved, and the results did not find significant effects on cognitive function (Andrieu et al., 2017). Despite these trials, several meta-analyses also analyzed multi-domain interventions on cognitive impairment. A Cochrane review found a small improvement in cognitive function with multi-domain interventions. However, whether multi-domain interventions could decrease dementia incidences was uncertain (Hafdi et al., 2021). Gavelin et al. (2021) reported that combined physical and cognitive training had a small beneficial effect on overall cognitive function in elder adults. Nutrition combined with physical exercise interventions could also improve global cognitive function in the elderly population (Liu et al., 2021).

## Conclusion

In conclusion, various pharmacological (cholinesterase inhibitors, memantine, antidiabetic agents, probiotics, cerebrolysin) and non-pharmacological interventions (cognition-oriented treatments, non-invasive brain stimulation physical exercise, and lifestyle-related interventions) have been proposed for cognitive impairment in older people. Although a variety of new drug targets has been identified for cognition enhancement in older adults, the new drug is still in development. The existing potential drug targets should be further exploited, and discovering new drug targets could be a solution to the lack of effective drugs. Most non-pharmacological interventions showed a small to moderate beneficial effect on cognitive function in cognitive impairment old people. Thus, combinations of pharmacological and non-pharmacological interventions or combinations of different types of non-pharmacological interventions may be more efficient in improving or preserving cognition.

## Author contributions

YZ designed and edited the review. LC searched the data and drafted the review. JJ searched the data. All authors contributed to the article and approved the submitted version.
